# Difference and relation between adolescents’ and their parents or caregivers’ reported oral health-related quality of life related to orthodontic treatment: a prospective cohort study

**DOI:** 10.1186/s12955-019-1094-0

**Published:** 2019-02-26

**Authors:** Katrien Jaeken, Maria Cadenas de Llano-Pérula, Jurgen Lemiere, Anna Verdonck, Steffen Fieuws, Guy Willems

**Affiliations:** 1 Department of Oral Health Sciences-Orthodontics, KU Leuven and Dentistry, University Hospitals Leuven, Kapucijnenvoer 7, 3000 Leuven, Belgium; 2 Child and Adolescent Psychiatry/Pediatric Haemato-oncology, University Hospitals Leuven, Herestraat 49, 3000 Leuven, Belgium; 3 Interuniversity Institute for Biostatistics and statistical Bioinformatics, KU Leuven and University Hasselt, Kapucijnenvoer 35, 3000 Leuven, Belgium

**Keywords:** Adolescents, Oral health-related quality of life, Parent-child agreement, Orthodontics, Prospective cohort study

## Abstract

**Background:**

Parents/caregivers play an important role in deciding whether their children will undergo orthodontic treatment or not. Their perceptions also have an influence on other choices involving orthodontic treatment. The purpose of this study was to investigate the difference and correlation between the ratings given by children and their parents or caregivers on their oral health-related quality of life (OHRQoL) before, during and after orthodontic treatment.

**Methods:**

In this ongoing observational prospective cohort study, 498 children aged 11 to 16 years-old and one of their parents/caregivers completed questionnaires before (T0), 1 year after start (T1) and 1 month after the end of orthodontic treatment (T2). OHRQoL was scored by using the Child Perception Questionnaire (CPQ_11–14_) and the Parental-Caregiver Perception questionnaire (P-CPQ). The self-perception of oral aesthetics was evaluated with the Oral Aesthetic Subjective Impact Scale (OASIS) in addition to the aesthetic component (AC) of the Index of Orthodontic Treatment Need (IOTN). Spearman correlations, Mann-Whitney U-tests and linear models were used to analyze the longitudinal data.

**Results:**

At T0, the ratings of parents/caregivers were significantly lower for the total CPQ as well as for the subdomains of oral symptoms, functional limitations and emotional well-being. Parents/caregivers also scored significantly lower at T2 for the total CPQ and the subdomain of oral symptoms. The relations between the scores of children and their parents/caregivers were significant at all three time points, as were the changes in scores, but all of them were at most moderate in size. Parents/caregivers scored significantly lower for OASIS than their children at all time points and only at baseline a significant, weak correlation was found.

**Conclusion:**

The reports of parents/caregivers should be seen as important complementary information in OHRQoL research.

**Trial registration:**

This study was approved by the Medical Ethical Commitee of the University Hospitals Leuven and the Katholieke Universiteit Leuven (ML5739), Leuven, Belgium, on the 12th of May of 2009, with the registration number S51642. All procedures performed are in accordance with the ethical standards of the institutional and/or national research committees and with the 1964 Declaration of Helsinki and its later amendments or comparable ethical standards.

## Background

Parents or their legal guardians (in this study we will use the term caregivers) play a very important role in decision making, regarding their child’s health and oral health and the use of healthcare services. Therefore it is important to determine how accurate their estimation of their children’s quality of life is. Throughout the years, several questionnaires have been developed to compare child-parent reporting [1–3]. These proxy-reporting tools have been studied in multiple healthcare disciplines and enable us to examine the extent to which parents' assessments match those of their children. The results from these, mostly cross-sectional studies are contradictory. Some studies indicate that parents/caregivers show low correlation with the quality of life ratings of their children [4–8], while others find evidence of moderate to high correlation [1, 9, 10]. This depends on which health domain of healthcare is being looked at. The lowest correlation has been found for more subjective domains concerning emotional and social well-being [11–13], the highest for more objective ones such as physical domains [14]. The extent of this correlation can also be different according to the analyzed dimensions within health-related quality of life. These proxy reports are also valuable to understand quality of life by offering a more complete picture of the child’s near environment [15]. The magnitude and direction of the changes in parent-child agreement can only be understood through longitudinal study designs and in the context of a certain treatment, but only a few longitudinal studies have been conducted concerning health-related quality of life [16–18] and none of them regards the Oral Health-Related Quality of Life (OHRQoL).

The decision to undergo orthodontic treatment or not is often taken by parents and caregivers, and their perceptions have an influence on other choices involving orthodontic treatment [19, 20] based on what they think is best for their child’s well-being. This perception of well-being or quality of life (QoL) is defined by the world health organization as: “The individuals’ perception of their position in life in the context of the culture and value systems in which they live and in relation to their goals, expectations, standards and concerns” [21]. The influence of oral and dental conditions on the QoL of an individual is called the OHRQoL.

In order to evaluate OHRQoL, several tools have been designed, such as the Child Perception Questionnaire CPQ_11–14_ [6, 22]. A parental version of this questionnaire, the Parental-Caregiver Perception questionnaire (P-CPQ) [23], can be used in parallel in order to study parent-child agreement on OHRQoL. The P-CPQ has 31 items in common with the CPQ_11–14_ and both questionnaires have good validity and reliability [23, 24]. Self-perception of oral aesthetics together with perceived orthodontic treatment need are also important factors to consider when looking at a child’s OHRQoL.

Whether parents/caregivers can adequately make an estimation of their children’s OHRQoL and self-perception of oral aesthetics, remains to be elucidated. To our knowledge, no studies have been conducted with a prospective or longitudinal study design. Measuring OHRQoL reported by both patients and caregivers before, during and after orthodontic treatment could allow us to detect its change over time and to analyze its possible relation with the stages of treatment. Measurements before treatment permit us to stablish a baseline to which the rest of the measurements are compared. Knowing patient’s and parent’s perception of OHRQoL can also help the practitioner in explaining the goals of treatment and in following the cases up. Therefore, the aim of this study was to investigate the correlation and differences between the ratings given by children and their parents/caregivers on OHRQoL and self-perception of oral aesthetics before, during and after orthodontic treatment.

## Methods

This observational prospective cohort study was conducted in the University Hospitals Leuven, Leuven, Belgium and is an extension of a previous study [25]. Data collection started after the approval of the study protocol by the Medical Ethical Commitee of the University Hospitals Leuven and the Katholieke Universiteit Leuven (ML5739) in 2009. Prior to data collection an informed consent was given in writing to the participants and their parents/caregivers. The participants were selected according to the following inclusion and exclusion criteria at the first consultation and were asked to complete questionnaires. For this baseline sample (T0), we excluded children whose medical anamnesis showed psychological problems, children who previously underwent or were still undergoing orthodontic treatment, children with craniofacial anomalies, children and parents who did not understand Dutch. Children had to be between 11 and 17 years old.

After baseline, if an orthodontic treatment of any kind was advised and the patient did actually start treatment, patients and their caregivers were also asked to fill in questionnaires at two other times: 1 year after start of treatment (T1) and 1 month after completion of treatment (T2). These time periods were chosen to assess the participant’s perception (1) around the time when the treatment was halfway through and (2) after brackets were removed, so that the patient had not yet got accustomed to the new situation. Children and parents were asked to fill in the questionnaires separately to avoid one influencing the other. To ensure that this happened correctly, parents were asked to fill in the questionnaires in a separate room while their children were having their appliances fitted. Once they finished, children were asked to fill in their questionnaires separately as well.

A total sample of 498 parents/caregivers-children couples was obtained, of which 313 and 202 couples filled in the questionnaires at both T1 and T2 respectively. Of the total sample size of 498, 223 patients were not yet at the end of treatment, 1 unfortunately passed away and 42 dropped out of treatment because of lack of compliance. The other 30 missing values came from patients and parents who did not complete the questionnaire at every interval. No sample size estimation could be made due to the variability of the gathered data knowing the present study was only one part of an ongoing study that started in 2009.

Questionnaires for OHRQoL evaluation were given to the children and parents/caregivers. The questionnaire used for the children was the CPQ_11–14_ [22]. This consists of 37 diverse questions to assess a child’s OHRQoL and its 4 subdomains, namely oral symptoms, functional limitations, social well-being and emotional well-being. Each question applies to the frequency that a certain development concerning the lips, jaws and teeth occurred in the last 3 months. The answer options were shown as a 5-point Lickert scale (‘never’, ‘one or two times’, ‘sometimes’, ‘often’, ‘every day or almost every day’). To calculate the total CPQ score, responses were scored from 0 to 4 and every item was summed per child. For the CPQ scores in the different subdomains the same procedure was used. With these CPQ scores, evaluation of both the general (total CPQ score) and specific OHRQoL (per subdomain) was possible. A higher CPQ score corresponds with a lower OHRQoL and vice-versa.

The P-CPQ [23], was given to parents and caregivers to be completed. It consists of 33 varied questions for them to assess their child’s OHRQoL and it covers the same 4 subdomains. The answers to these questions have the same build-up as the CPQ_11–14_, and scores are counted, summed and interpreted in the same way.

Further, the Oral Aesthetic Subjective Impact Scale (OASIS) [26] was used for children to assess, and for parents/caregivers to estimate their child’s self-perception of oral aesthetics. The questionnaire consists of five questions concerning the perceptions of the patients themselves and what they think the perceptions of others are. Answers are scored on a 7-point Lickert scale: the higher the OASIS score the higher the aesthetic concern. Perceived orthodontic treatment need was evaluated by patients and parents or caregivers by using the aesthetic component (AC) of the index of orthodontic treatment need (IOTN) [27]. They evaluated the patient’s occlusion by matching the dental appearance of the patient to one of the photographs.

### Statistical analysis

The change in scores of the AC of the IOTN, the CPQ and OASIS indexes between T0, T1 and T2 was assessed by comparison between the scores obtained from children and parents using a multivariate linear model for longitudinal measures. An unstructured covariance matrix was used for the 6 measurements per child (3 from the child, 3 from the parent). Since a direct likelihood approach is used, all observations are included in the analysis, i.e. also from subjects with one or more missing measurements. Correlations between scores from children and parents were derived from this multivariate normal model, more specifically the correlation between the scores and changes in score of the child and parent at each time point. The 95% confidence interval (CI) for the correlation was based on the Fisher’s transformation of the correlation coefficient [28]. All analyses were performed using SAS software, version 9.4 of the SAS System for Windows.

## Results

A total of 498 children-caregiver couples were included in the study sample at T0. At T1 and T2, results for OHRQoL, AC and OASIS, of 313 and 202 couples were obtained respectively. The mean duration of the orthodontic treatment was 32.9 months.

**Fig. 1:**
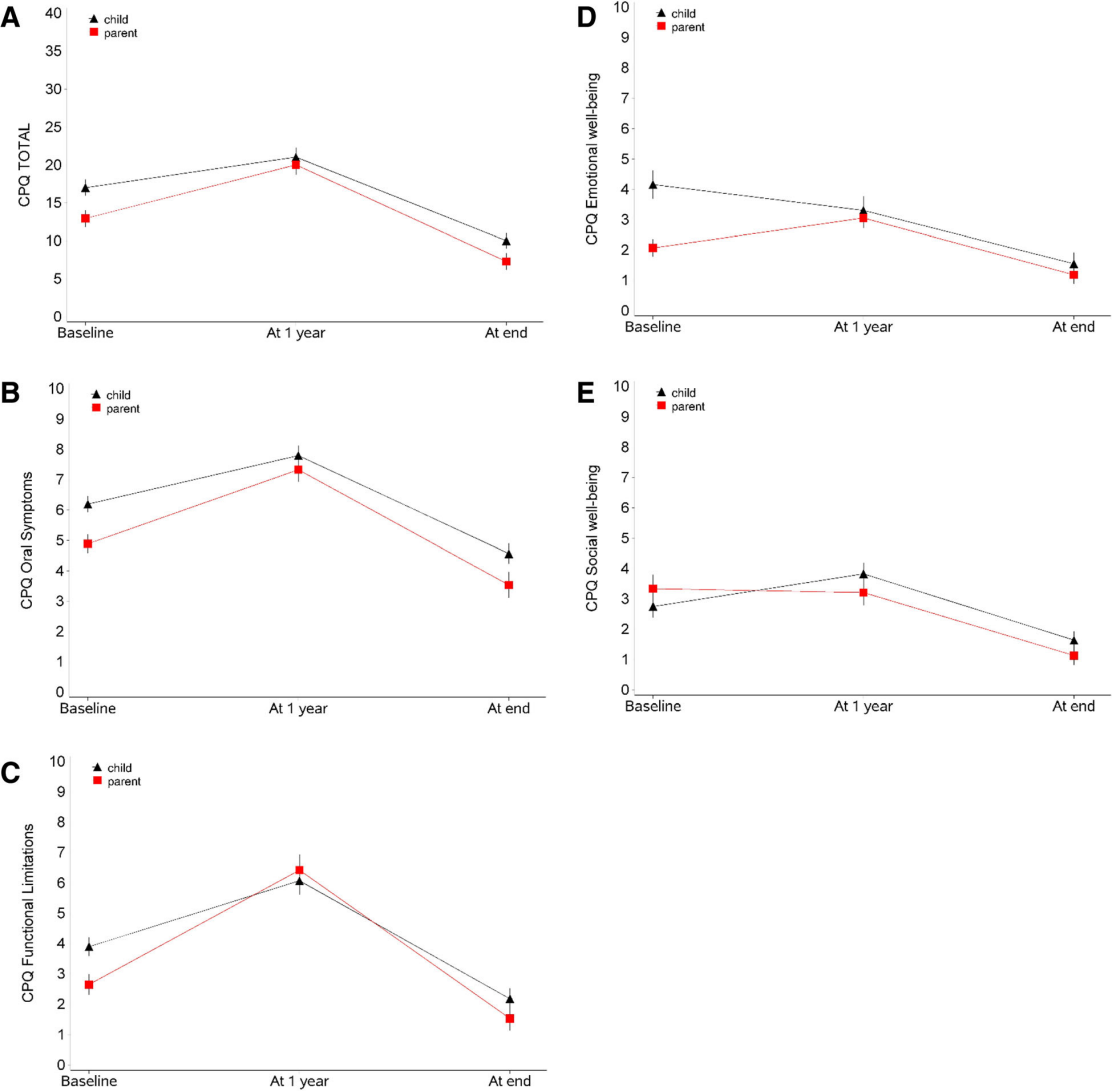
Results of the Child Perception Questionnaire and the Parental-Caregiver Perception Questionnaire. (**a**) Results for the total CPQ. For parents/caregivers (red line): difference between all timepoints with *P* < 0.0001. (**b**) Results for the subdomain of oral symptoms. For parents/caregivers (red line): difference between all timepoints with *P* < 0.0001. (**c**) Results for the subdomain of functional limitations. For parents/caregivers (red line): difference between all timepoints with *P* < 0.0001. (**d**) Results for the subdomain of emotional well-being. For parents/caregivers (red line): difference between all timepoints with *P* < 0.0001 (**e**) Results for the subdomain of social well-being. For parents/caregivers (red line): difference between all timepoints with *P* < 0.0001, except for T1-T2 *P* = 0.99

The results of the Parental CPQ showed a similar evolution compared to the results obtained from their children, except for the subdomains of social and emotional well-being (Fig. 1). The total P-CPQ and its subdomains of oral symptoms, functional limitations and emotional well-being (Fig. 1 A-D) showed a significant increase (*P* < 0.0001) from T0 to T1 and a significant decrease (*P* < 0.0001) from T1 to T2 and T0 to T2 (*P* < 0.0001). In the subdomain of social well-being (Fig. 1E) there is a significant decrease (*P* < 0.0001) from T1 to T2 and from T0 to T2, but no evidence for a change between T0 and T1. The results of the children’s CPQ were described in detail in a previous article of our group [25].

When the scores of children and parents are compared (Table 1) a significant difference between both is visible at T0 for the scores of total CPQ and the subdomains of oral symptoms, functional limitations and emotional well-being. At T1, no significant differences were found, while at T2, a significant difference was reported only for total CPQ and the subdomain of oral symptoms. Parents/caregivers consistently reported significantly lower CPQ scores at T0, compared to their child at T0 and T2, except for the subdomain of social well-being. The same significant trends were found at T2 for the total OHRQoL and the subdomain of oral symptoms.

**Table 1:** Differences (95% confidence intervals) in scores between parents and children at each time point

	Difference (95%Cl)	*P*-value
CPQ oral symptoms		
Baseline	1.31 (1.00;1.61)	<.0001*
After 1 year	0.47 (0.03;0.91)	0.2929
1 month after treatment	1.03 (0.59;1.47)	<.0001*
CPQ functional limitations		
Baseline	1.25 (0.89;1.62)	<.0001*
After 1 year	−0.35 (− 0.92;0.22)	0.8379
1 month after treatment	0.65 (0.20;1.10)	0.0506
CPQ emotional well-being		
Baseline	2.09 (1.66;2.52)	<.0001*
After 1 year	0.25 (−0.26;0.77)	0.9294
1 month after treatment	0.36 (−0.04;0.77)	0.4863
CPQ social well-being		
Baseline	−0.59 (−1.06;-0.12)	0.1406
After 1 year	0.62 (0.16;1.08)	0.0852
1 month after treatment	0.51 (0.15;0.86)	0.0569
CPQ total		
Baseline	4.06 (2.96;5.15)	<.0001*
After 1 year	1.02 (−0.41;2.44)	0.7243
1 month after treatment	2.72 (1.57;3.87)	<.0001*
Treatment need (AC)		
Baseline	2.47 (2.26;2.69)	<.0001*
After 1 year	1.28 (1.11;1.45)	<.0001*
1 month after treatment	0.22 (0.05;0.39)	0.1073
OASIS with AC		
Baseline	6.15 (5.61;6.69)	<.0001*
After 1 year	4.12 (3.57;4.68)	<.0001*
1 month after treatment	2.23 (1.59;2.87)	<.0001*
OASIS without AC		
Baseline	3.70 (3.25;4.15)	<.0001*
After 1 year	2.82 (2.33;3.31)	<.0001*
1 month after end	2.02 (1.44;2.59)	<.0001*

A positive difference refers to a higher mean score for parents. *P*-value = *p*-value after Tukey correction for multiple testing (* = *p* < 0.05)

Significant positive correlations were found between children and parents for the CPQ results at T0, T1 and T2, and also for the changes in CPQ scores (Table 2). The results show that the strength of the relation was at most moderate (all correlations being lower than 0.50).

**Table 2:** Correlation between scores and changes in scores of parents/caregivers and children at each time point

	T0		T1		T2		T1-T0		T2-T0		T2-T1	
	Rho (Cl)	*P*-value	Rho (Cl)	*P*-value	Rho (Cl)	*P*-value	Rho (Cl)	*P*-value	Rho (Cl)	*P*-value	Rho (Cl)	*P*-value
CPQ oral symptoms	0.4275 (0.3530;0.4966)	< 0.0001*	0.3184 (0.2155;0.4142)	< 0.0001*	0.3875 (0.2653;0.4974)	< 0.0001*	0.2788 (0.1748;0.3768)	< 0.0001*	0.4344 (0.3249;0.5324)	< 0.0001*	0.2827 (0.1449;0.4098)	< 0.0001*
CPQ functional limitations	0.3578 (0.2789;0.4320)	< 0.0001*	0.3063 (0.2021;0.4037)	< 0.0001*	0.3187 (0.1910;0.4359)	< 0.0001*	0.3326 (0.2291;0.4286)	< 0.0001*	0.2832 (0.1641;0.3942)	< 0.0001*	0.1922 (0.0602;0.3176)	0.0046*
CPQ social well-being	0.3441 (0.2644;0.4192	< 0.0001*	0.3586 (0.2583;0.4512)	< 0.0001*	0.3334 (0.2048;0.4506)	< 0.0001*	0.2019 (0.0974;0.3020)	0.0002*	0.2786 (0.1563;0.3926)	< 0.0001*	0.2490 (0.1244;0.3658)	0.0001*
CPQ emotional well-being	0.4321 (0.3579;0.5008)	< 0.0001*	0.2190 (0.1134;0.3196)	< 0.0001*	0.3322 (0.2064;0.4471)	< 0.0001*	0.1839 (0.0798;0.2841)	0.0006*	0.2735 (0.1506;0.3880)	< 0.0001*	0.1802 (0.0530;0.3017)	0.0057*
CPQ total	0.4821 (0.4118;0.5467)	< 0.0001*	0.3829 (0.2854;0.4725)	< 0.0001*	0.4302 (0.3120;0.5353)	< 0.0001*	0.3006 (0.1972;0.3975)	< 0.0001*	0.4121 (0.2969;0.5154)	**< 0.0001**	0.2714 (0.1446;0.3894)	< 0.0001*
Treatment need AC	−0.0267 (−0.1142;0.0612)	0.5515	0.0992 (−0.0106;0.2066)	0.0765	0.0853 (−0.0484;0.2160)	0.2107	−0.0324 (−0.1394;0.0752)	0.5549	−0.0065 (− 0.1275;0.1146)	0.9160	− 0.0309 (− 0.1706;0.1101)	0.6684
OASIS with AC	0.1470 (0.0601;0.2318)	0.0010*	0.1280 (0.0189;0.2341)	0.0216	0.0458 (−0.0887;0.1787)	0.5049	0.1173 (0.0101;0.2217)	0.0320	0.0787 (−0.0558;0.2104)	0.2509	0.0964 (−0.0436;0.2327)	0.1765
OASIS without AC	0.1564 (0.0697;0.2408)	0.0005*	0.0786 (−0.0312;0.1865)	0.1603	0.0184 (−0.1161;0.1522)	0.7891	0.1363 (0.0291;0.2403)	0.0129	0.0929 (−0.0412;0.2237)	0.1740	0.0943 (−0.0450;0.2299)	0.1841

CI: 95% confidence interval based on Fishers transformation of correlation coefficient. * Statistically significant *P*-values

The evolution of children’s self-perception of their oral aesthetics has also been previously reported in detail by our group [25]. The present study looked at how parents/caregivers evaluated the impact of malocclusion on their children and its evolution. For parents, mean values of the AC and OASIS both with and without AC remained constant from T0 to T1 to T2 (*p* > 0.1). Compared to their children, parents scored the AC of their children significantly lower at T0 and T1 (Table 1). For OASIS, parents’ results were significantly lower at T0, T1 and T2 (Table 1). Here, no correlations between parents and their children were found, except for OASIS at baseline (Table 2), where only a weak significant correlation was detected.

## Discussion

The present study assesses the relation between the reports of parents and their children on oral health related quality of life. To our knowledge, this is the only observational prospective cohort study performed before, during and after orthodontic treatment. While no correlation could be found at T0, results showed a moderate correlation for the total CPQ score at T1 and T2 between parents and children, as well as for the changes in results over time. The correlation was also similar in all subdomains of CPQ. We analyzed the covariation between the ratings of parents and their children using Pearson coefficients. These coefficients only indicate the correlation between scores but they give no indication of ‘absolute agreement’. However, previous literature has focused mainly on the caregiver-child agreement in OHRQoL ratings. A moderate to low agreement has been demonstrated between proxies and their children before undergoing orthodontic treatment [5, 6, 8, 29, 30] while other studies report a moderate to low agreement during orthodontic treatment [31, 32]. This differences regarding the level of agreement generally depend on the health domain in which quality of life is being looked at. Discrepancies can be due to differences in perspectives, but also to a lack of insight from parents/caregivers into their children’s lives and social worlds. Parents can have a limited knowledge of their children’s relationships and activities outside the family home and how do they affect their internal feelings [12, 13]. Therefore, when assessing the OHRQoL of orthodontic patients, both perceptions of children and their parents/caregivers should be considered as complementary, as otherwise valuable information might be lost [6, 31]. The correlations found in this study were at most moderate and some of them were only weak. Note also that the absence of a strong relation between the results from children and parents can also be partially due to the reliability of the scores, due to the inherent limitations of self-rating. Performing measurement error could therefore attenuate the observed relations.

For the self-perception of oral aesthetics, a low agreement was found only at T0. The impact of this finding is relatively unknown since the agreement was never evaluated in previous research related to OHRQoL. An explanation may be that these items are difficult to observe and parents are less capable of judging these internalized problems accurately [15]. However, oral aesthetic impact of malocclusion appears to be important in children’s motivation for orthodontic treatment [26], which is the reason why we used the OASIS scale in spite of having limited testing of reliability in comparison with other indexes like the CPQ.

Results from the present study also revealed that at T0 are significantly higher except for the subdomain of social well-being. At T2, parents only rate significantly higher than their children concerning the total CPQ and the subdomain of oral symptoms. Concerning the ratings on perception of oral aesthetics, parents’ scores were significantly lower at all times. In literature, different outcomes have been discussed. Some studies show that children experience a greater negative impact of their OHRQoL than the one reported by their parents regarding total CPQ score, functional limitations, social well-being [29] and oral symptoms [30, 33, 34]. On the other hand, studies conducted during treatment with fixed appliances, show that adolescents reported no significant differences in OHRQoL scores compared to their parents/caregivers in the domains of emotional well-being, social well-being and total CPQ, while finding a significantly higher negative impact reported by the children for the oral symptoms subscale [31, 32, 35]. This in direct contrast with the results of the present study, where no significant difference between parents and their children was found at T1. A possible explanation for this can be that the questionnaire at each time point is the same, making parents more aware of the importance of OHRQoL the second time they are asked, although contradictorily, a significant difference is seen again at T2.

Although the general differences between reports of parents and their children were statistically significant in our study, the magnitude of this difference was small, suggesting that both reports are appropriate to use as complementary measure for evaluation of OHRQoL, because they potentially highlight different aspects.

For interpretation of our results, some limitations of the study design need to be taken into consideration. The sample of participants was taken only from the University Hospitals Leuven, where patients were often already referred by the general dentist due to a manifest malocclusion. This setting will probably not be representative for the Belgian population, as patients from private practices were not included. Also there was substantial missing information due to lack of compliance in the study.

Further, the children included at baseline were 11 to 16 years old and the CPQ_11–14_ questionnaire was used, which was defined for a lower and upper age limit of respectively 11 and 14 years of age. The same questionnaire was also used 2 to 3 years later, after treatment. Besides, no control group couples, with randomization between experimental and control group, were included because of ethical considerations. Also, while very similar, the CPQ and the P-CPQ scales do not have the same number of items. In our study, comparisons were made on the difference between the overall score per item, in order to respect the integrity of each scale, but comparison only on the common points could constitute a valid approach as well.

On the other hand, this study has several considerable strengths. The major relevant feature of this study is the prospective study design and follow up. There are to our knowledge no previous studies comparing adolescent’s reports and proxy reports on OHRQoL and self-perception of oral aesthetics, related to orthodontic treatment, with a prospective study design. All our findings resulted from OHRQoL measures that have shown good validity and reliability [23, 24]. Further, the original validated questionnaires in English were translated to Dutch and no norm values were validated for Dutch speakers. However, we only studied the differences, correlations and changes. Therefore the potential impact of the fact that there was no Dutch validation can be somehow disregarded.

## Conclusion

In general, parents and caregivers report a higher OHRQoL of their children, especially before starting orthodontic treatment. During the whole timeline of treatment there seems to be a moderate correlation between the scores of parents and children. Therefore, it is preferable to obtain data directly from children whenever possible. Parents’ reports should be seen as complementary information, which can be especially relevant at the initial stages of diagnosis and treatment planning, because their perceptions can have an influence on choices involving orthodontic treatment.

## Abbreviations

AC: Aesthetic Component
CPQ_11–14_: Child Perception Questionnaire
IOTN: Index of Orthodontic Treatment Need
OASIS: Oral Aesthetic Subjective Impact Scale
OHRQOL: Oral Health-Related Quality of Life
P-CPQ: Parental-Caregiver Perception Questionnaire
QoL: Quality of life
T0: baseline
T1: 1 year after start or orthodontic treatment
T2: 1 month after the end of orthodontic treatment
